# Deep Learning-Derived Pathomic Features Predict NCIT Efficacy in Resectable Locally Advanced ESCC: Clinical Utility and Mechanistic Insights

**DOI:** 10.3390/curroncol33030136

**Published:** 2026-02-26

**Authors:** Kunrui Zhu, Jie Tong, Yaqi Duan, Yiming Li, Yanqi Feng, Yuelin Han, Xiangtian Xiao, Zhuoyan Han, Shu Xia

**Affiliations:** 1 Institute of Oncology, Tongji Hospital, Tongji Medical College, Huazhong University of Science and Technology, Wuhan 430030, China; d202482566@hust.edu.cn (K.Z.); m202576789@hust.edu.cn (J.T.); liymtj@hust.edu.cn (Y.L.); d202382250@hust.edu.cn (Y.F.); u201910354@hust.edu.cn (Y.H.); m202376505@hust.edu.cn (X.X.); m202476651@hust.edu.cn (Z.H.); 2 Institute of Pathology, Tongji Hospital, Tongji Medical College, Huazhong University of Science and Technology, Wuhan 430030, China; yqduan@hust.edu.cn

**Keywords:** pathomics, neoadjuvant chemoimmunotherapy, machine learning, esophageal squamous cell carcinoma

## Abstract

**Background**: Esophageal squamous cell carcinoma (ESCC) is the predominant subtype of esophageal cancer, with poor outcomes following neoadjuvant chemoradiotherapy (NCRT). Neoadjuvant chemoimmunotherapy (NCIT) has emerged as a promising strategy, but reliable predictive biomarkers remain lacking. This study aimed to develop an AI-driven pathomic model for NCIT response prediction and explore its biological mechanisms. **Methods**: We analyzed 269 H&E-stained whole-slide images (WSIs) from 198 ESCC patients (104 from Tongji Hospital, 94 from TCGA). Using ResNet152, we segmented WSIs into four tissue categories (tumor cells, stroma, lymphocytes, and necrosis), extracted spatially weighted pathomic features, and constructed the ECiT score via logistic regression. An integrated model combining the ECiT score with clinical variables (T stage, P53 status) was developed. Mechanistic analyses were performed using TCGA-ESCA and GSE160269 datasets. **Results**: The integrated model achieved AUCs of 0.897 (training) and 0.809 (temporal validation), outperforming clinical (AUC = 0.624) and pathomic-only (AUC = 0.751) models. Mechanistically, a high ECiT score correlated with enhanced immune activation (elevated CD4^+^ memory T cell infiltration), while low scores were linked to endoplasmic reticulum (ER) stress-unfolded protein response (UPR) activation. EIF2S3 was identified as a key molecular mediator, correlating with three pathomic features, UPR activation, and poor prognosis. **Conclusions**: This study may offer a preliminary indicator that could assist in personalized clinical decision-making. Correlative evidence suggests that the EIF2S3-mediated ER stress–UPR axis represents a potential candidate therapeutic target to overcome NCIT resistance, generating testable hypotheses to advance precision oncology for resectable locally advanced ESCC.

## Simple Summary

Esophageal squamous cell carcinoma exhibits high mortality and limited therapeutic options. While immune checkpoint inhibitors improve outcomes, identifying non-responders to neoadjuvant chemoimmunotherapy remains urgent. This study developed a predictive model for treatment efficacy using deep learning and real-world cohort data, with mechanism exploration via TCGA datasets. Integrating histopathological images and clinical variables, the model demonstrated a robust performance and revealed associations between treatment response, immune activation, and specific cellular processes. These findings offer insights that may inform personalized therapeutic strategies and improve the understanding of potential mechanisms underlying immunotherapy resistance.

## 1. Introduction

Esophageal cancer (EC) ranks as the seventh most common cause of cancer-related mortality globally, characterized by high lethality, frequent diagnosis at advanced stages, and limited treatment options [1]. Esophageal squamous cell carcinoma (ESCC) represents the predominant subtype, accounting for approximately 88% of all EC cases [2]. For locally advanced EC (stage T1-4N1-3M0), neoadjuvant chemoradiotherapy (NCRT) followed by surgical resection has long served as the standard treatment paradigm [3,4]. However, nearly half of patients experience postoperative disease progression [5], underscoring the urgent need for more effective therapeutic strategies to improve outcomes.

The advent of immune checkpoint inhibitors (ICIs) has revolutionized the management of advanced EC [6,7,8,9]. In the neoadjuvant setting, neoadjuvant chemoimmunotherapy (NCIT) have also demonstrated promising efficacy in ESCC, with pathological complete response (pCR) rates ranging from 15.4% to 59%, significantly higher than those achieved with neoadjuvant chemotherapy alone [10,11]. A retrospective study further confirmed superior 2-year overall survival (OS) and disease-free survival (DFS) with NCIT compared to conventional NCRT [12]. Notably, single-cell transcriptomic studies have shed light on the mechanistic basis for ESCC’s relative sensitivity to immunotherapy: ESCC harbors a distinct tumor microenvironment (TME) characterized by enriched tumor-infiltrating lymphocytes (TILs), higher density of tertiary lymphoid structures (TLSs), and coordinated anti-tumor immune cell interactions—features that distinguish it from less responsive subtypes like esophageal adenocarcinoma (EAC) [13]. Despite these advancements, a substantial subset of patients exhibits poor response to NCIT [14], highlighting the critical need for reliable predictive biomarkers, specifically for NCIT response, to guide individualized therapeutic decisions and optimize treatment efficacy.

Pathomics, an emerging discipline that leverages advanced computing technologies to extract and analyze quantitative features from pathological images—encompassing cell morphology, tissue structure, and spatial arrangements—has become a powerful tool in precision medicine [15]. Enabled by breakthroughs in artificial intelligence (AI), particularly deep learning (DL), pathomics has transformed histopathological image analysis, a process central to cancer diagnosis, subtype classification, prognosis prediction, and treatment response assessment [16,17]. Recently, AI-driven pathological analysis has become increasingly integral to EC management, with applications spanning from early lesion detection to prognosis stratification and treatment response prediction, addressing key limitations of traditional subjective histopathological evaluation, such as inter-observer variability and the incomplete capture of tumor heterogeneity [18]. Importantly, AI-guided quantification of H&E-stained slides has already been validated for assessing TILs and TLS density in ESCC [13], providing a non-invasive means to characterize TME features associated with immunotherapy response, findings that underscore the clinical translatability of AI-driven pathomics in EC.

To date, pathomics has proven to be a valuable tool in research across various cancers [19,20,21]. Furthermore, the utility of pathomics in predicting immunotherapy efficacy has been validated across multiple cancer types, from identifying responders in lung and gastric cancers [22,23] to forecasting pathological complete response in colorectal [24,25] and breast cancers [26], highlighting its broad applicability in precision medicine. In EC, preliminary AI-based pathomics studies have shown considerable promise: Yang et al. [27] developed a convolutional neural network (CNN) model that quantifies nuclear morphology in pathological images to identify five stages of ESCC tissue transformation with 99% accuracy, while Chen et al. constructed a DL model based on biopsy hematoxylin-eosin (H&E) sections to predict chemotherapy efficacy and prognosis in ESCC [28]. Notably, Li et al. [29] recently developed a DL pathomics system (ESCC-PS) that outperforms PD-L1 expression in predicting progression-free survival (PFS) following immunotherapy for ESCC, achieving an accuracy of 84.5% in the validation cohort and a combined model area under the curve (AUC) of 0.904 when integrated with PD-L1, underscoring the potential of pathomics as a robust prognostic tool in immunotherapy settings. Single-cell studies have further identified key TME components predictive of immunotherapy response in ESCC, including CXCL13^+^CD8^+^ T cells and CXCL9^+^CXCL10^+^ tumor-associated macrophages (TAMs), whose coordinated interactions drive interferon-γ-dominated immune activation [13]. These cell-type-specific signatures have been validated to predict ICB response across multiple cohorts with AUC > 0.7 [13], providing a biological framework for pathomic marker development. However, studies focusing on pathological response prediction to NCIT remain scarce.

In recent years, the development of foundation models has enhanced the generalization capabilities of AI-based pathological analysis models, enabling adaptation to image data from diverse institutions and scanning devices [30]. Nevertheless, these models often rely on large-scale public datasets for training and lack validation in specific clinical scenarios, such as NCIT response prediction for ESCC [20]. Furthermore, the “black box” nature of many AI models limits their clinical translatability, as the biological interpretability of pathomic features remains inadequate—an issue highlighted as a key barrier to integrating AI into routine EC management [31]. Thus, we aim to develop a model to identify the TME features associated with high response during NCIT in ESCC patients and further construct a comprehensive prediction model based on biopsy WSIs to assist in the decision-making of NCIT regimens for ESCC. Additionally, we conducted a mechanistic exploration of the image biomarker using the ESCA dataset from The Cancer Genome Atlas (TCGA) and GSE160269 from the Gene Expression Omnibus (GEO) database, with the aim of uncovering its underlying biological mechanisms and providing a biological explanation for the marker, thereby offering references for precision medicine.

## 2. Materials and Methods

### 2.1. Patients and Dataset

**Figure 1:**
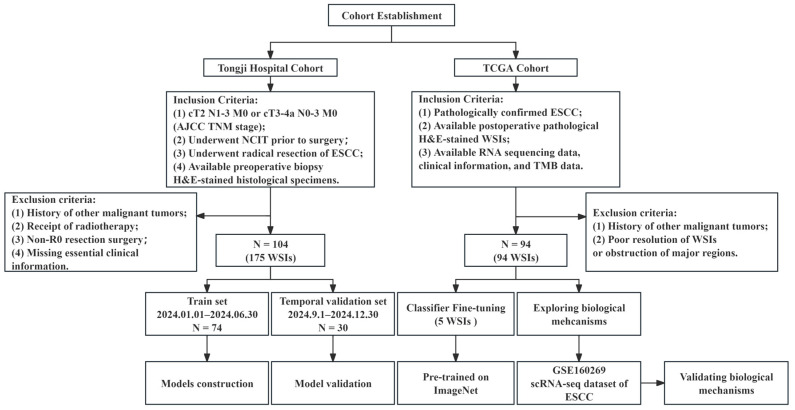
Composition of the study cohort (N: number of cases).

A total of 269 H&E-stained whole-slide images (WSIs) from 198 patients with ESCC were analyzed. Among these, 104 consecutive cases (visiting Tongji Hospital, Tongji Medical College, Huazhong University of Science and Technology from January to December 2024) were assigned to the model development cohort (Figure 1), with chronological dataset division of 74 patients admitted before June for model development and 30 admitted after September for temporal model validation. All patients in this cohort had resectable disease and received NCIT prior to radical esophagectomy. Only patients who underwent R0 radical esophagectomy were included, while those with R1 or R2 resection were excluded to avoid confounding from heterogeneous adjuvant treatments (e.g., postoperative radiotherapy) and ensure reliable evaluation of major pathologic response. A small subset of patients received adjuvant immunotherapy postoperatively due to N2 stage or failure to achieve major pathological response. Retrospectively collected clinicopathological data (from the hospital’s electronic database) included age, gender, smoking history, initial clinical TNM stage, neoadjuvant immunotherapeutic agents, and treatment cycles. The 1-year RFS rate was determined by re-examination imaging results during outpatient follow-up until November 2025. Histological diagnosis was confirmed by two experienced pathologists via independent review of preoperative biopsy and postoperative specimens. Major pathologic response (MPR) was defined as residual viable tumor cells < 10% in the tumor bed. All analyzed WSIs were pathologist-screened to include representative tumor and tumor microenvironment regions. This retrospective study was approved by the Institutional Ethics Committee of Tongji Medical College, Huazhong University of Science and Technology.

The molecular mechanism exploration cohort included 94 ESCC cases from TCGA and 64 ESCC cases from GSE160269. Pathologists conducted quality control on open source WSIs, excluding those of poor quality. All data were retrieved from the GDC portal (https://portal.gdc.cancer.gov/) under the TCGA-ESCA project, including RNA-seq data (transcriptome profiling, gene expression quantification), clinical information (raw XML files with detailed records), WSIs (imaging data, diagnostic slides in formats such as .svs), and SNP (simple nucleotide variation) data. These files were added to the cart and downloaded via GDC-client (v2.3.0) with commands specifying directories and tokens, then systematically organized in compliance with TCGA data usage policies and ethical guidelines.

### 2.2. WSIs Preprocessing

The H&E-stained slides from our center were digitized using a digital pathological scanner (Ningbo Jiangfeng Bio-information Technology Co., Ltd., Ningbo, China) at a 20× equivalent magnification, corresponding to a pixel resolution of 0.5 μm/pixel. All WSIs underwent tissue region detection via the Otsu thresholding algorithm to segregate tissue areas from the background. Deep learning-based algorithms were employed for automatic segmentation of WSIs into 256 × 256 pixel patches, with only those exhibiting > 70% tissue coverage retained. Additional filtering procedures were applied to exclude patches containing motion blur, staining artifacts, or other technical imperfections. Subsequent to patch extraction, color normalization preprocessing (Vahadane algorithm [32]) was applied to mitigate batch effects arising from variations in staining protocols and scanner parameters.

First, 6080 preprocessed image patches from 5 ESCC cases in TCGA were annotated by pathologists into four categories: ESCC cell regions (ESCCs), cancer-associated stroma (CAS), tumor-infiltrating lymphocytes (LYMs), and necrotic regions (DEBs). These annotated images were subsequently split into training and test subsets (7:3 ratio) to facilitate fine-tuning of the ResNet152 classification model. The last fully connected layer of ResNet152 was modified during construction to adjust its output dimension to 4, corresponding to four target categories. The remaining layers of the model maintain the pretraining weights during the initial training period to take advantage of their learned generic feature representations. The CrossEntropyLoss function was used as the optimization objective, and the stochastic gradient descent (SGD) optimizer was used to update the parameters. The learning rate was set to 0.001, and the momentum parameter was set to 0.9. After training, the classification performance of the model was evaluated on the validation set and test set, respectively. The main evaluation index was accuracy, which was the proportion of correctly classified samples to the total number of samples.

### 2.3. Pathomic Feature Selection and Model Construction in Tongji Hospital Cohort

#### 2.3.1. Case-Level Patches Feature Extraction and Fusion

To analyze the spatial correlation of the tumor microenvironment (TME), relative spatial distances were calculated separately for patches in each of the four classified categories (ESCCs, CAS, LYM, and DEB). Specifically, for the k-th patch in category c of case i (denoted as *P_i_**_,c,k_***), its Euclidean distance to the tumor center (defined as the geometric centroid of manually annotated ESCCs regions) was computed as *d_i,c,k_*. Image patches located closer to the tumor center are assigned higher weights during feature fusion; here, a normalized inverse distance weighting (IDW) strategy was adopted to quantify this weight, with the weight of *P_i,c,k_* calculated as:

(1) $$w_{i,c,k}=\frac{\frac{1}{d_{i,c,k+\epsilon }}}{\sum_{k=1}^{K_{i,c}}(\frac{1}{d_{i,c,k+\epsilon }})}$$

where i represents total number of cases, ***c*** represents patch categories, and *K**_i,c_*** represents the number of patches in category c of case i. In addition, pathological experts performed quality control on the selected images of each category to ensure their representativeness of the corresponding cases (only patches with no ambiguous tissue boundaries or excessive background noise were retained). These qualified patches were then batch-input into the ResNet152 model as a feature extractor, where the final fully connected layer of the original architecture was removed, and the 2048-dimensional feature vector output by the penultimate layer was retained to characterize the deep visual features of the four screened patch categories, denoted as *f**_i,c,k_*** for *P**_i_*****_,_*****_c,k_***. To obtain the category-level fused feature, the weighted average of patch-level features was computed using the distance-dependent weights in Equation (1), resulting in the 2048-dimensional fused feature vector for category c of case i:

(2) $$F_{i,c}=\sum_{k=1}^{K_{i,c}}w_{i,c,k}\cdot f_{i,c,k}$$

Finally, stacking the four category-specific fused vectors row-wise yielded the 4 × 2048-dimensional case-level feature matrix *F**_i_***, which integrates both deep visual features and spatial context of the TME.

#### 2.3.2. Explore the Pathomic Signatures of Different NCIT Responses

We employed a two-stage feature selection method. Firstly, the maximum relevance and minimum redundancy (mRMR) algorithm was used to eliminate the features that were irrelevant to or redundant with the NCIT response [33]. Subsequently, the lasso logistic regression combined with 5-fold cross-validation was adopted to select the most predictive features. After two-step feature selection, we integrated four types of pathological image-derived features into seven machine learning classifiers, random forest (RF), K-nearest neighbor (KNN), logistic regression (LR), extreme gradient boosting (XGBoost), naive Bayes (NB), decision tree (DT), and support vector machine (SVM), to construct a pathomic signature (ECiT score) for NCIT response prediction. In this study, we used the SHAP analysis to interpret the contributions of the pathomic features to NCIT responses.

Univariate and multivariate logistic regression analyses were conducted to examine the associations between clinical variables and NCIT efficacy. Clinical features with a univariate *p* value < 0.05 were retained for subsequent development of the clinical model. Missing values were addressed through imputation. An integrated model was constructed by combining the ECiT score with preselected clinical variables (*p* value < 0.05 in univariate analysis), following the identical methodological framework as the clinical model, including consistent data partitioning, standardization, class balancing, regularization scheme, and hyperparameter optimization procedures.

Feature importance of each predictive model was analyzed using the absolute values of logistic regression coefficients to clarify the contribution weight of each feature to prediction outcomes. Continuous prediction probabilities were converted into binary results by determining the optimal threshold via maximizing the Youden index. The primary evaluation metric was the AUC, with classification performance further analyzed using confusion matrices. For the multimodal feature model, a nomogram was constructed to visually quantify the contribution of each feature to MPR probability. Additionally, decision curve analysis (DCA) was performed to assess the clinical utility of the model by comparing net benefits across different threshold probabilities. External validation was conducted using operative and biopsy images from 30 independent cases to verify the model’s generalizability.

### 2.4. Mechanism Explanation in the TCGA and GEO Dataset

In the TCGA cohort, feature extraction followed the same protocol as the Tongji cohort to maintain consistent image feature profiles. The pre-established Model B, initially trained and validated on the Tongji cohort, was transferred to the TCGA cohort for classification. TCGA cases were divided into immune-activated and immune-tolerant groups based on predicted immunotherapy sensitivity, enabling model generalization across cohorts. Transcriptomic differential analysis between the two groups leveraged TCGA’s RNAseq data using DESeq2 for normalization and differential expression calculation. Gene Ontology (GO) enrichment analysis (covering biological processes, cellular components, and molecular functions) and Kyoto Encyclopedia of Genes and Genomes (KEGG) pathway analysis were carried out using standard bioinformatics tools. Terms with *p* < 0.05 after multiple testing corrections were considered significantly enriched. The false discovery rate (FDR) was applied for multiple testing corrections. Gene Set Enrichment Analysis (GSEA, v4.3.3) was employed to perform hallmark pathway enrichment analysis across different ECiT score groups. Additionally, the mean expression of key differentially expressed genes between the two groups was visualized, and enrichment was conducted based on expression differences in four feature categories to explore gene-correspondent, high-dimensional image feature correlations.

**Figure 2:**
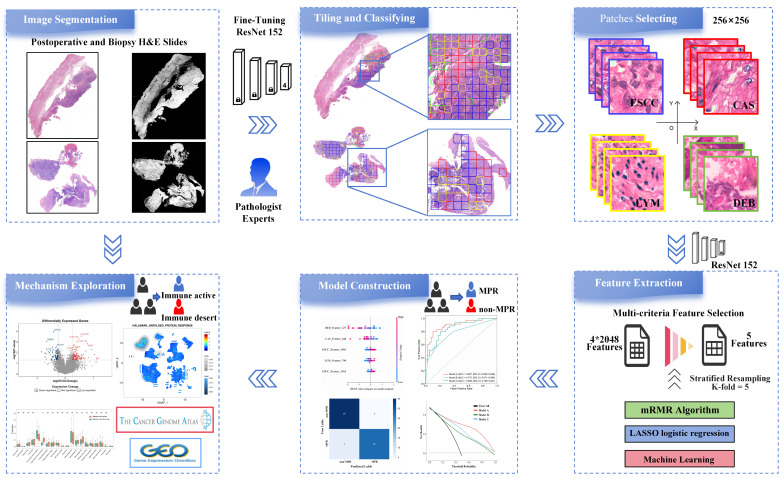
Analytical pipeline for pathological image analysis. The pipeline consists of four key steps: WSIs preprocessing, feature extraction, model development, and biological mechanism exploration (ns: not significant; *: *p* < 0.05; and ***: *p* < 0.001).

The ESTIMATE algorithm quantified immune cell infiltration by computing immune and stromal scores in the tumor microenvironment, with intergroup differences statistically tested. TCGA’s SNP data analyzed tumor mutational burden (TMB) differences, calculated as somatic non-synonymous mutations per megabase. Finally, OS analyses evaluated prognostic differences between the two groups in TCGA. Kaplan–Meier survival curves and log-rank tests determined the statistical significance of survival disparities. Cell clustering and annotation of GSE160269 single-cell datasets were performed using the Seurat tool [34]. Additionally, the pathway activity of each cell was calculated via the AddModuleScore method in Seurat (v4.1.0), followed by visualization using UMAP. The entire analytical pipeline for pathological image analysis in this study, including WSIs preprocessing, feature extraction, model development and biological mechanism exploration, is summarized in Figure 2.

### 2.5. Statistical Analysis

Baseline characteristic comparisons: Continuous variables were tested for normality using the Shapiro–Wilk test. Normally distributed data were compared using independent samples *t*-tests, and non-normally distributed data using the Mann–Whitney U test. Categorical variables were compared using the χ^2^ test or Fisher’s exact test. Analyses were performed using Python 3.6 (Anaconda3, v23.11.0), with core libraries including scipy (for statistical tests), scikit-learn (for model development and metrics), and lifelines (for survival analysis). A fixed random state (42) was used to ensure reproducibility. All statistical tests were two-tailed, with a significance level set at *p* < 0.05.

## 3. Results

### 3.1. Clinical Characteristics of the Cohort

Following the predefined inclusion and exclusion criteria (Figure 1), a total of 104 patients with ESCC were consecutively enrolled from Tongji Hospital, who were subsequently divided into the model discovery (training) and validation cohorts. The overall cohort had a mean age of 63.76 ± 6.63 years, with a male predominance (80.77%) and a smoking history reported in 69.23% of cases. After neoadjuvant chemotherapy and immunotherapy (NCIT), 58 patients (55.77%) failed to achieve a major pathological response (non-MPR), whereas 46 patients (44.23%) attained MPR. The analysis of the distribution of demographic and clinicopathological variables shows that no differences are observed in all the listed clinical characteristics between the two datasets (Table 1). The results of the differential analysis on the clinical characteristics between the MPR and non-MPR groups showed that patients with a more severe depth of tumor invasion (T3 vs. T2) and those with P53 mutation had a significantly higher proportion of non-MPR compared to patients with wild-type P53, and the differences were statistically significant (Table S1). Due to the short follow-up period, only three patients were observed to have local recurrence, all of whom were non-MPR patients, and no tumor-related deaths occurred in the total cohort.

### 3.2. Construction of Pathomics Signatures Based on Different NCIT Responses

**Figure 3:**
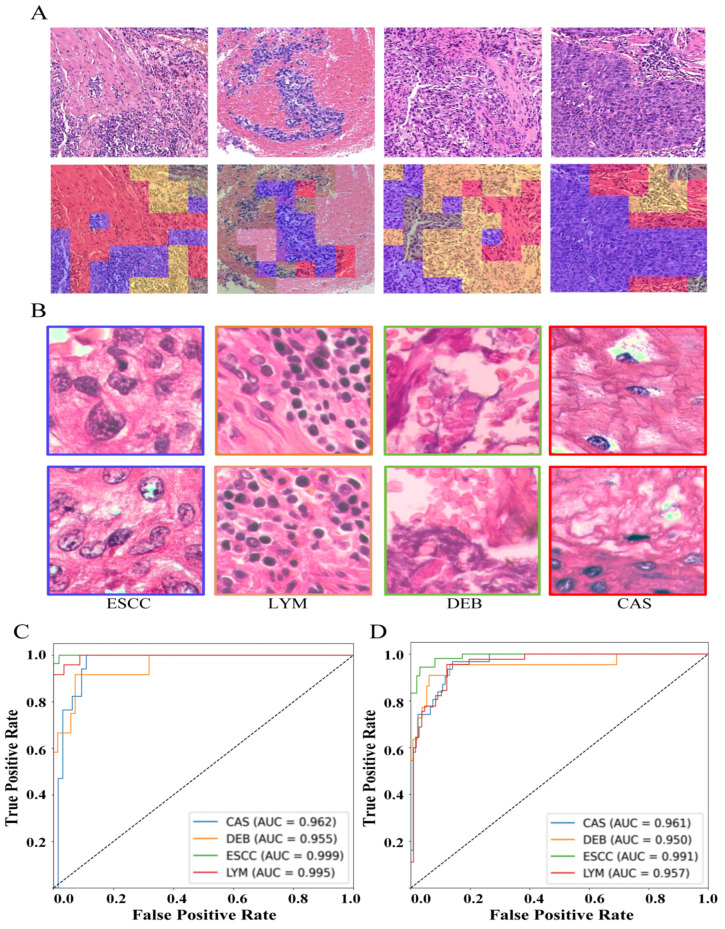
Pathological image classification: Visualization and evaluation. (**A**) (**Top**): Magnified WSIs (2048 × 2048); (**bottom**): Corresponding 4 classification heatmaps (256 × 256 blocks: red = CAS, blue = ESCCs, yellow = LYM, and green = DEB; uncolored areas = non-target features/blank); (**B**) representative 256 × 256 images of the four classes; (**C**) ROC curves of fine-tuned ResNet152 (4-class task) on the test set; and (**D**) ROC curves of the model in the validation set.

To address the automatic classification of four distinct histopathological image categories (CAS, DEB, ESCC, and LYM), a transfer learning framework was implemented using the pre-trained ResNet152 architecture. Local interpretability visualization of the model’s classification outputs (Figure 3A) illustrates the spatial attention patterns corresponding to each category. Representative histopathological images of the four classes, alongside the model’s predicted segmentation maps, are presented in Figure 3B. Quantitative evaluations demonstrated robust performance: on the independent test set, the model achieved class-specific AUC values of 0.962 (CAS), 0.955 (DEB), 0.999 (ESCC), and 0.995 (LYM), with an average AUC of 0.978 (Figure 3C). On the validation set, the corresponding class-wise AUCs were 0.961 (CAS), 0.950 (DEB), 0.991 (ESCC), and 0.957 (LYM), yielding an average AUC of 0.965 (Figure 3D).

**Figure 4:**
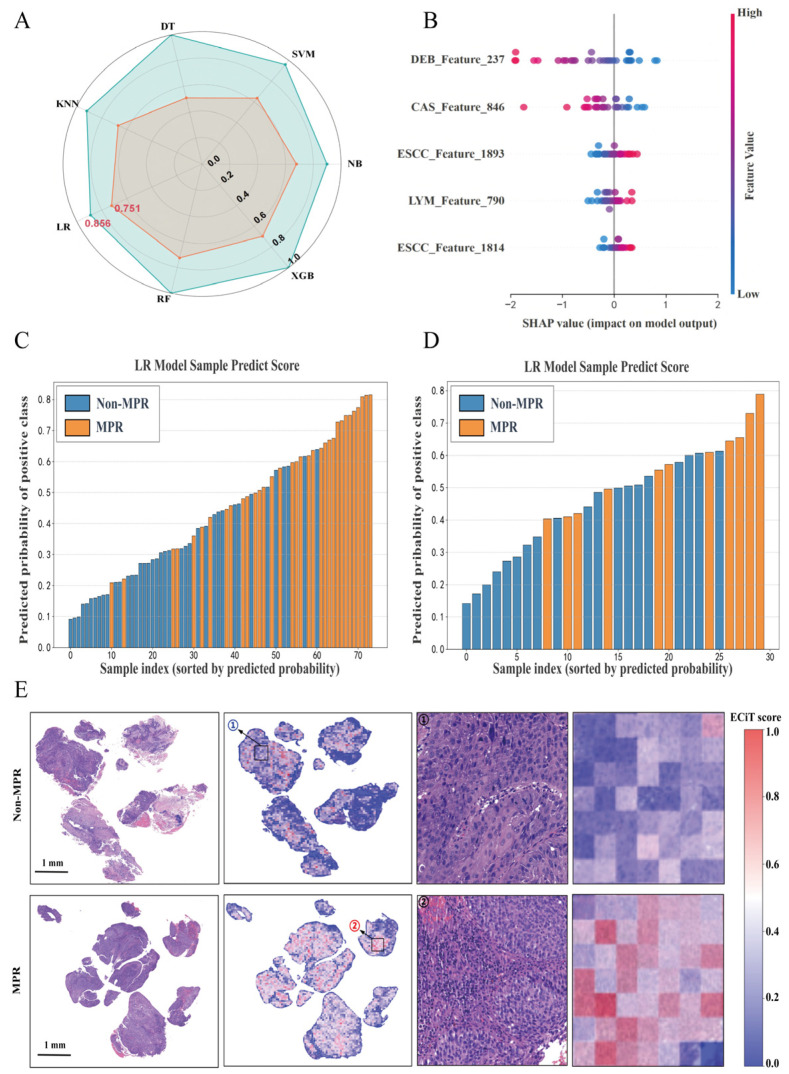
Pathological feature-driven model construction and validation for NCIT response prediction. (**A**) Radar plot of AUC values across seven machine learning models: the green polygon represents training dataset performance, and the orange polygon denotes temporal validation dataset performance (DT: decision tree; SVM: support vector machine; XGB: extreme gradient boosting; RF: random forest; KNN: k-nearest neighbors; and NB: naive Bayes); (**B**) SHAP summary plot of the 5 pathological features used for model construction, showing their impact on ECiT score output; (**C**,**D**) predicted response probability (y-axis) of the feature-derived model in the training dataset (**C**) and temporal validation dataset (**D**), stratified by sample index (sorted by probability, x-axis); MPR cases (orange) cluster in higher-probability strata; (**E**) WSIs (scale bar = 1 mm) and corresponding ECiT score heatmaps for non-MPR (**top**) and MPR (**bottom**) cases; Region ① and region ② represent representative local magnifications of the non-MPR and MPR cases’ images, respectively, with corresponding ECiT score visualizations (histology and heatmap insets); The MPR samples show dense feature distribution aligned with elevated scores.

Following a two-stage feature selection process, five pathological features (ESCC_1814, ESCC_1893, CAS_846, LYM_790, and DEB_237) per case were ultimately identified and employed for the construction of the machine learning models. To assess the predictive performance of models for NCIT response, we evaluated seven machine learning algorithms using key metrics (AUC, accuracy, recall rate, and F1 score) on both training and internal validation cohorts (Table S2). In the training set, several models (e.g., NB, XGB, and DT) achieved a perfect AUC of 1.000, which may suggest potential overfitting to the training data. In contrast, the logistic regression (LR) model achieved a more moderate AUC of 0.856. Critically, on the internal validation set, LR achieved the highest AUC (0.751) among all models, outperforming alternatives such as NB (0.727) and the support vector machine (SVM, 0.636; Table S2). The radar plot (Figure 4A) visually illustrates the stability of AUC performance across training and validation sets. For secondary metrics in the validation cohort, LR also delivered a competitive performance: its accuracy of 0.700 was the highest among all models, while its F1 score (0.471) surpassed those of models like XGB (F1 = 0.333) and DT (F1 = 0.250; Table S2). Collectively, the LR model outperforms the other six algorithms in generalizability and predictive efficacy for immunotherapy response, with minimal overfitting risk (relative to models with perfect training AUCs). Thus, we selected the LR model as the foundation for developing the ECiT score.

To assess the discriminative stability of the ECiT score for treatment response stratification, we performed validation in a temporal cohort. The ECiT score demonstrated consistent predictive performance across cohorts: the area under the curve (AUC) was 0.978 (95% confidence interval [CI], 0.950–1.000) in the training cohort and 0.863 (95% CI, 0.744–0.982) in the temporal validation cohort. To contextualize feature contributions to model output, SHAP (SHapley Additive exPlanations) analysis was conducted (Figure 4B): among the five pathological features included in model construction, DEB_237 and CAS_846 exhibited the strongest positive impact on predicted response probability, while ESCC_1814, ESCC_1893, and LYM_790 contributed to reduced predicted probability, with ESCC_1814 showing the most pronounced negative effect. When patients were ranked by their predicted response probability (derived from the ECiT score), responders (MPR) consistently clustered in the higher-probability strata across both the non-MPR (Figure 4C) and MPR (Figure 4D) subgroups, confirming the score’s ability to stratify response risk. Representative pathological images (Figure 4E) further illustrated distinct spatial patterns of the ECiT score between non-MPR and MPR cases: MPR samples displayed a denser, more heterogeneous distribution of the signature features (visualized in the right-hand heatmap), consistent with the elevated ECiT scores observed in responders.

### 3.3. Performance of the Integrated Models in Predicting NCIT Response

**Figure 5:**
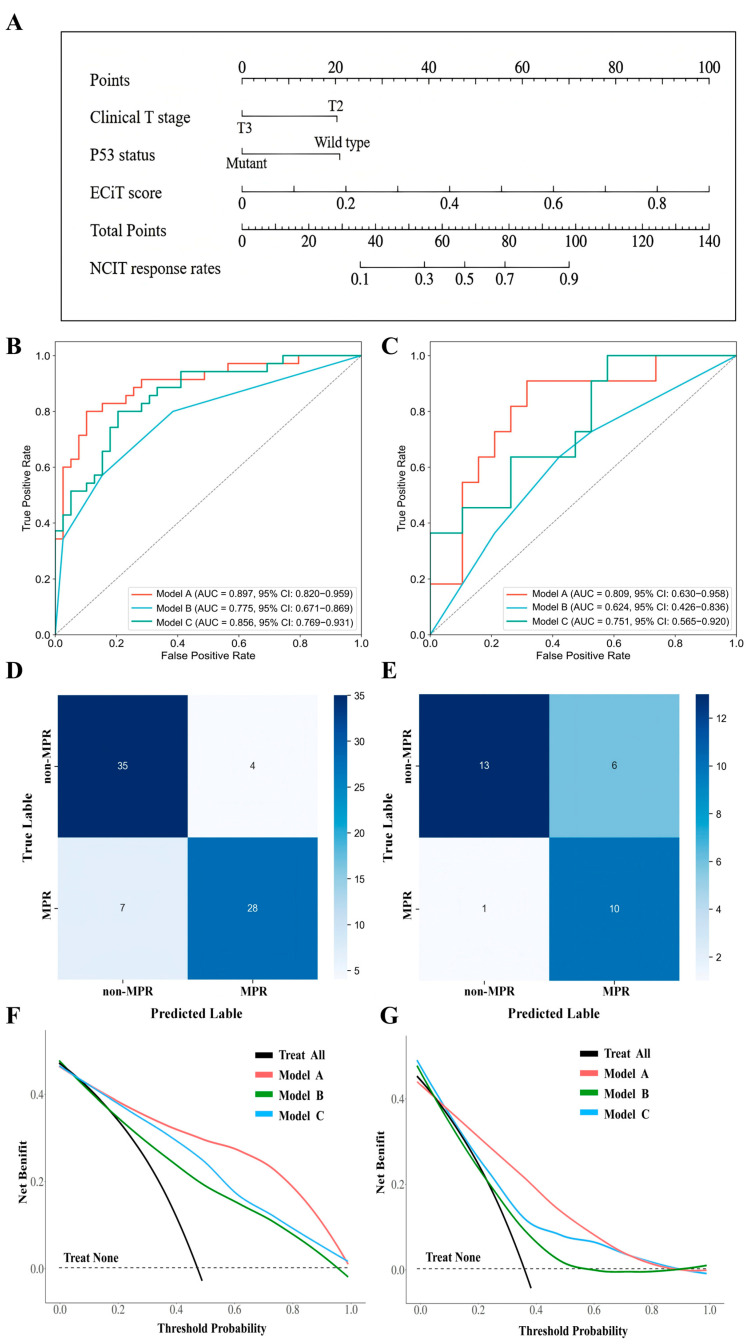
Development, validation, and clinical utility of an integrated model for NCIT response prediction. (**A**) Nomogram for NCIT response prediction, integrating clinical T stage, P53 status, and ECiT score; weighted points map to predicted response probability. (**B**,**C**) Calibration curves ((**B**): training set; (**C**): validation set) showing alignment between the integrated model’s predicted MPR probability and actual responses. (**D**,**E**) Confusion matrices ((**D**): training set; (**E**): validation set) stratifying predicted vs. actual MPR/non-MPR cases (color = case count). (**F**,**G**) DCA curves ((**F**): training set; (**G**): validation set) comparing net benefit of the integrated model (Model A), clinical feature model (Model B), and pathological image model (Model C); Model A shows superior utility across threshold probabilities.

Univariate logistic regression revealed that the initial clinical T3 stage was significantly associated with a reduced likelihood of NCIT response compared to T2 (*p* = 0.007; OR = 0.2695% CI CI: 0.10–0.69; Supplementary Table S3). Similarly, the pretreatment clinical N2 stage correlated with a lower probability of NCIT response versus N0-1 (*p* = 0.016; OR = 0.19,95% CII: 0.05–0.73). The P53 mutation was significantly linked to decreased NCIT response likelihood relative to the wild type (*p* < 0.001; OR = 0.14, 95%CI: 0.05–0.41). No significant associations with NCIT response were observed for sex, pretreatment clinical stage, smoking history, ICI types, or treatment cycles (all *p* > 0.05). A logistic clinical prediction model was constructed by incorporating variables with *p* < 0.05. Ultimately, T stage and P53 status were included in the Model B (Table S3). To assess multicollinearity among independent variables, variance inflation factors (VIFs) were calculated. Results showed that, in combined Model C, the VIF values for ECiT score and P53 status were 1.20 and 1.18, respectively. The distribution of features in Model C (pathomics feature model) across different NCIT response groups in the Tongji hospital cohort can be seen in Supplementary Figure S1. All VIF values were substantially below the threshold of 5, indicating no significant multicollinearity in the models. To further enhance predictive performance for NCIT response, we constructed an integrated logistic regression model (Model A) by combining clinical variables (T stage, P53 status) with the ECiT score. The relative contribution of each predictor to Model A is visualized in the nomogram (Figure 5A), where weighted points for each variable sum to a total score that maps to the predicted NCIT response probability.

We compared the performance of Model A (integrated), Model B (clinical features only), and Model C (pathological image features only) across training and temporal validation datasets. In the training set (Figure 5B), Model A achieved the highest AUC (0.897, 95% CI: 0.820–0.959), outperforming Model B (AUC = 0.775, 95% CI: 0.671–0.869) and Model C (AUC = 0.856, 95% CI: 0.769–0.931). In the temporal validation set (postoperative pathomic feature input; Figure 5C), Model A retained superior performance (AUC = 0.809, 95% CI: 0.630–0.958), while Model B (AUC = 0.624, 95% CI: 0.426–0.836) and Model C (AUC = 0.751, 95% CI: 0.565–0.920) exhibited diminished generalizability. Quantitative metrics (Table S4) confirmed Model A’s robustness: In the training set, it achieved an accuracy of 0.851, sensitivity of 0.800, and F1 score of 0.836; in the validation set, these metrics were 0.767, 0.909, and 0.741, respectively. Notably, Model A balanced sensitivity and specificity more effectively than Model B (validation set accuracy = 0.600) or Model C (validation set specificity = 0.421). Confusion matrix analysis (Figure 5D,E) further elucidated the classification performance of Model A. In the training cohort (Figure 5D), Model A correctly classified 35 non-MPR cases and 28 MPR cases, yielding only 4 false positives and 7 false negatives. In the temporal validation cohort (Figure 5E), Model A maintained robust classification performance, with 13 true non-MPR cases, 10 true MPR cases, 6 false positives, and 1 false negative. Decision curve analysis (DCA; Figure 5F,G) further demonstrated Model A’s clinical utility: across both training and validation datasets, Model A yielded a higher net benefit than Model B, Model C, and “treat all”/”treat none” strategies across a broad range of threshold probabilities. Collectively, these results confirm Model A as the optimal tool for NCIT response prediction.

### 3.4. Mechanistic Exploration of Pathomic Signatures in the TCGA Dataset

**Figure 6:**
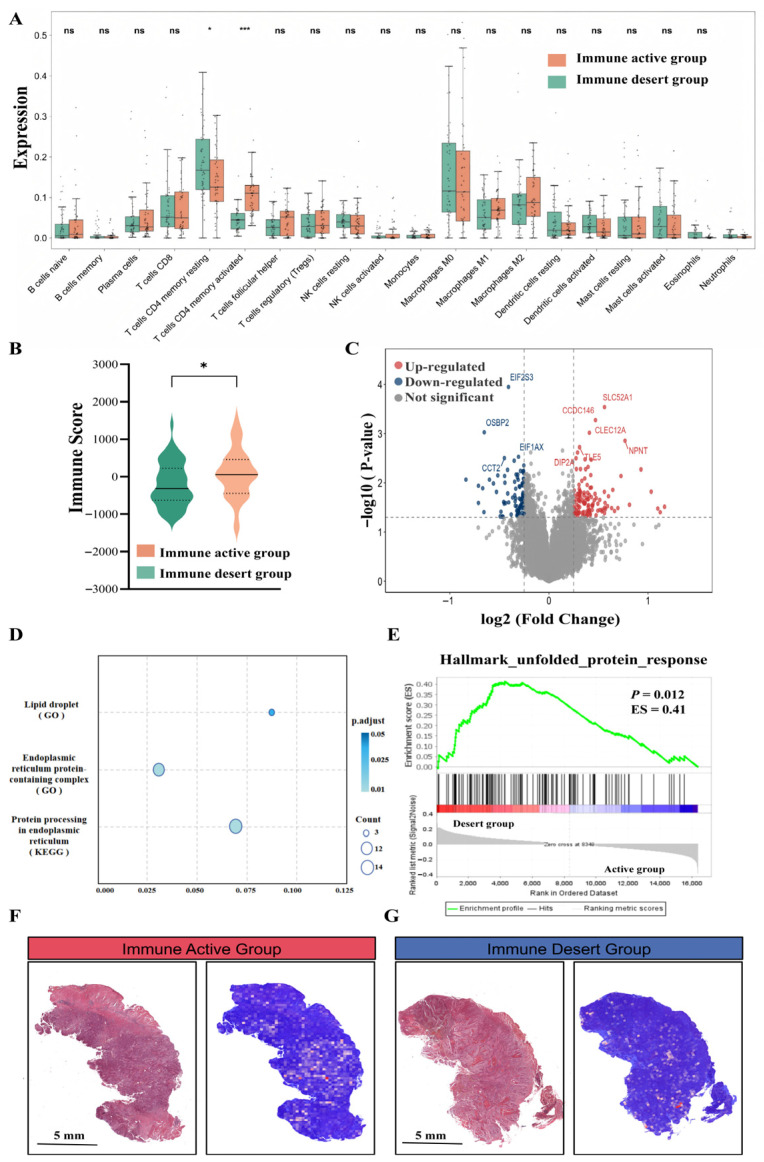
Immune microenvironment and transcriptomic profiles stratified by ECiT score in TCGA_ESCC dataset. (**A**) Immune cell infiltration levels (CIBERSORT) in immune-active (orange) vs. desert (green) groups (ns: not significant; *: *p* < 0.05; and ***: *p* < 0.001); (**B**) boxplot of ESTIMATE immune scores (*: *p* < 0.05); (**C**) volcano plot of DEGs between high/low ECiT score subgroups (*p* < 0.05, |log_2_FC| > 0.2); (**D**) GO/KEGG enrichment of DEGs; (**E**) GSEA plot of the Hallmark Unfolded Protein Response pathway (enriched in the desert group, *p* = 0.012, and ES = 0.41); and (**F**,**G**) ECiT score visualization (red = high, blue = low) in representative TCGA WSIs.

To investigate the biological implications of the pathological image features in the ECiT score, we first transferred this LR model to the TCGA cohort to stratify cases by immunotherapy response potential. Key distributions of the ECiT score across this cohort are depicted in Supplementary Figure S2; the optimal stratification threshold (0.19) determined via Youden’s index to maximize diagnostic discrimination was used to stratify TCGA cases into an immune-active group (ECiT score ≥ 0.19, *n* = 43) and an immune-desert group (ECiT score < 0.19, *n* = 51). CIBERSORT immune cell infiltration analysis (Figure 6A) revealed that the immune-active group exhibited a significantly higher infiltration of CD4^+^ memory T cells (*p* < 0.001) but lower infiltration of neutrophils (*p* = 0.03) and resting CD4^+^ T cells (*p* = 0.02) compared to the immune-desert group. Analysis of SNP data showed no significant difference in TMB between the two groups (*p* = 0.31; Table S5), and Kaplan–Meier survival analysis also did not identify a difference in overall survival (OS; *p* = 0.98; Figure S3). ESTIMATE analysis (Figure 6B) demonstrated that the immune-active group had a significantly higher immune score than the immune-desert group (81.62 ± 648.70 vs. −200.96 ± 584.70; *p* = 0.03), though no significant difference was observed in stromal scores (*p* = 0.07; Figure S4).

Transcriptomic differential analysis between high and low ECiT score subgroups identified 326 differentially expressed genes (DEGs; *p* < 0.05, |log_2_ fold change| > 0.20; Figure 6C). Gene Ontology (GO) enrichment analysis of these DEGs revealed significant enrichment in lipid droplet (LD) metabolism (upregulated in the immune-active group; *p* = 0.04) and endoplasmic reticulum (ER)-associated protein complex pathways (upregulated in the immune-desert group; *p* = 0.01; Figure 6D). KEGG pathway analysis further confirmed enrichment of protein processing in the ER (upregulated in the immune-desert group; *p* = 0.01; Figure 6D). GSEA (Figure 6E) showed that the Hallmark Unfolded Protein Response (UPR) pathway, a component of ER-associated protein complex signaling, was significantly enriched in the immune-desert group (*p* = 0.012, enrichment score [ES] = 0.41). Finally, visualization of ECiT scores in representative TCGA WSIs (Figure 6F,G) showed that WSIs with higher ECiT scores (immune-active group) exhibited denser TILs infiltration, as confirmed by pathological expert review.

**Figure 7:**
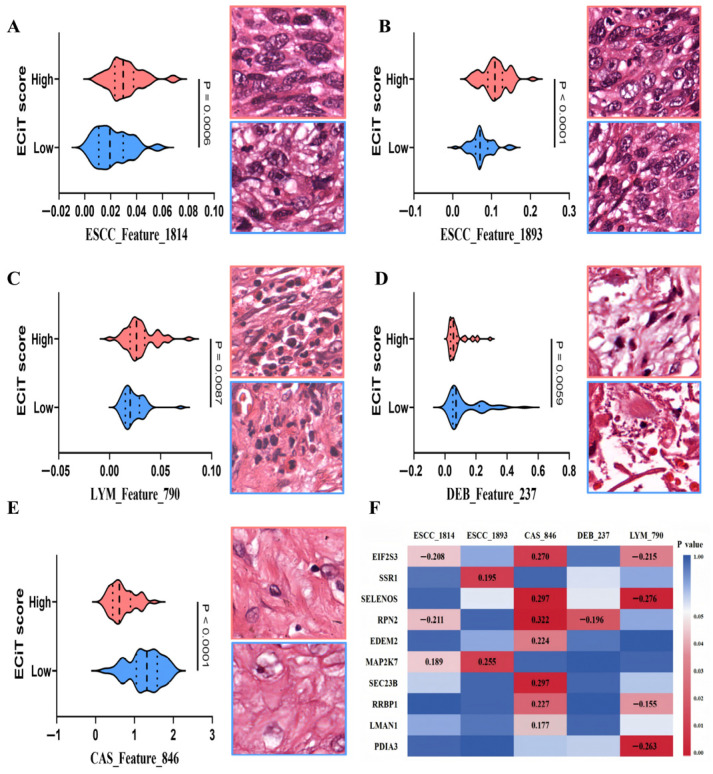
Morphological and molecular correlates of pathological image features with the ECiT score. (**A**–**E**) Correlation of patches’ features with ECiT score and representative image patches; (**F**) heatmap of ER/UPR pathway top 10 DEG correlations with the five features; and EIF2S3 and RPN2 correlate with three features (number: correlation coefficient; color: *p*-value: right bar).

To further elucidate the biological correlates of the five pathological image features, we clustered image patches by their feature scores (using the previously established grouping strategy) and had a board-certified pathologist perform a single-blind assessment, where the pathologist was blinded to the ECiT score grouping of each patch of consistent morphological traits across each cluster. Correlation analysis revealed that three features (ESCC_1814, ESCC_1893, and LYM_790) exhibited a positive correlation with the ECiT score (Figure 7A–C): Higher ESCC_1814 scores corresponded to image patches with greater tumor nuclear density (Figure 7A); elevated ESCC_1893 scores were associated with a higher proportion of tumor cells with nuclear-cytoplasmic vacuoles and increased cytoplasmic transparency (Figure 7B); and higher LYM_790 scores aligned with patches showing greater lymphocyte nuclear density and a higher likelihood of adjacent vascular distribution (Figure 7C).

Conversely, CAS_846 and DEB_237 showed a negative correlation with the ECiT score: Lower DEB_237 scores were linked to more prominent inflammatory responses in image patches (Figure 7D); reduced CAS_846 scores corresponded to patches with looser, irregularly arranged fibrous tissue components (Figure 7E). Among the features, CAS_846 was associated with the largest number of differential genes (e.g., EIF2S3, SELENOS, and RPN2), followed by LYM_790. Notably, EIF2S3 and RPN2 expression correlated with the scores of three distinct pathological image features. In particular, EIF2S3 showed unique associations with ESCC, CAS, and LYM features, suggesting its potential central regulatory role. Further analysis of the protein–protein interaction network (Figure S5) indicated that, compared with the other nine candidate genes, EIF2S3 exhibited stronger interactions with core components of the UPR pathway (EIF2S1, ATF4, HSPA5, EIF2AK3, and DDIT3). These observations support that EIF2S3 may serve as a more critical hub involved in UPR-mediated regulation of TME immune function, and we therefore focused subsequent in-depth analyses on EIF2S3. Collectively, these findings link the pathological image features of ECiT score to distinct immune microenvironment and transcriptomic profiles, particularly ER/UPR signaling and TIL infiltration, validating the biological relevance of the ECiT score.

**Figure 8:**
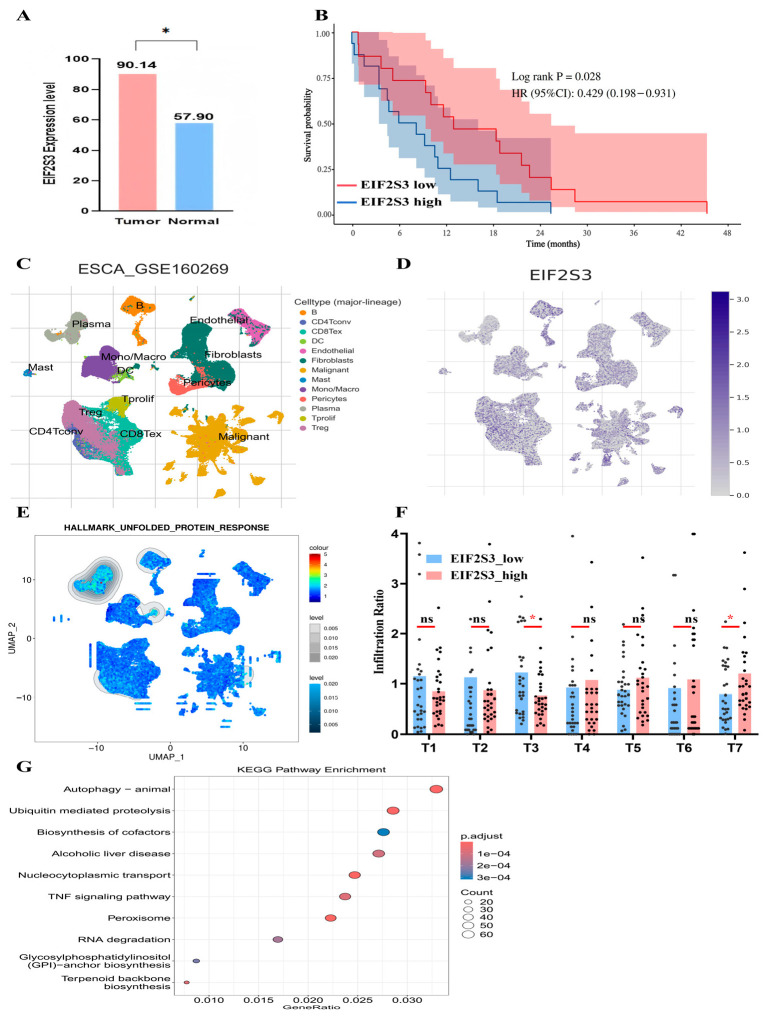
EIF2S3 expression, clinical prognosis, and functional correlates in ESCC. (**A**) EIF2S3 expression levels in tumor vs. normal tissues (*: *p* < 0.05); (**B**) Kaplan–Meier survival curve: EIF2S3-high patients have worse overall survival (log-rank *p* = 0.028, HR = 0.429, and 95% CI: 0.198–0.931); and (**C**) UMAP of single-cell clusters (GSE160269) in esophageal cancer. Clusters include malignant cells, immune cells, and stromal cells. (**D**) EIF2S3 expression distribution in single-cell clusters. (**E**) UMAP of UPR pathway activity; activity correlates with EIF2S3 expression (analyzed via TISCH: http://tisch.comp-genomics.org/). (**F**) Immune cell infiltration ratios in EIF2S3-high vs. low groups (T1: Effector CD8^+^ T cell; T2: Effector CD4^+^ T cell; T3: HSP-high T cell; T4: IFN-high T cell; T5: Exhausted CD8^+^ T cell; T6: Naive/memory T cell; T7: Regulatory T cell; ns: not significant; * *p* < 0.05). (**G**) KEGG pathway enrichment of EIF2S3-associated genes.

EIF2S3 encodes a eukaryotic translation initiation factor, and our findings reveal its close associations with pathological image features and TME immune function. Analysis of TCGA data showed that EIF2S3 expression was significantly higher in ESCC tumor tissues than in adjacent normal tissues (Figure 8A). Clinical prognostic analysis demonstrated that patients with high EIF2S3 expression had significantly worse overall survival (log-rank *p* = 0.028, HR = 0.429, and 95% CI: 0.198–0.931; Figure 8B), identifying EIF2S3 as a potential prognostic biomarker. We also performed Kaplan–Meier survival analysis for the remaining nine candidate genes (e.g., RPN2, SELENOS, and PDIA3). The results showed that only PDIA3 exhibited a significant prognostic association (log-rank *p* = 0.037; Table S7). Notably, PDIA3 displayed less centrality in the UPR network compared with EIF2S3 (Figure S5) despite its strong correlation with the LYM feature. To further validate the crosstalk between EIF2S3, the UPR pathway, and TME immunity, we leveraged the GSE160269 cohort to map EIF2S3 expression across cell types: EIF2S3 was predominantly expressed in malignant cell clusters and CD4^+^ T cells (Figure 8C,D). Concomitantly, the UPR pathway (a core endoplasmic reticulum [ER] stress pathway) exhibited elevated activity in EIF2S3-high cell subsets (Wilcoxon rank-sum test, *p* < 0.01; Figure 8E), confirming the link between EIF2S3, ER/UPR signaling, and CD4^+^ T cells. Using T cell subset markers from a recent ESCC single-cell study (seven subsets defined in Table S6 [13]), we further found that EIF2S3 expression negatively correlated with the infiltration level of HSP-high T cells (a kind of Effecting T cell) and positively correlated with Treg cell exhaustion (*p* < 0.05, Figure 8F). Finally, KEGG pathway enrichment analysis of EIF2S3-associated genes revealed enrichment in autophagy, ubiquitin-mediated proteolysis, the TNF signaling pathway, and other ER stress-related pathways (Figure 8G).

## 4. Discussion

This study innovatively integrates pathomic features extracted from H&E-stained WSIs with clinical variables to establish a robust predictive model for evaluating the efficacy of NCIT in ESCC. The results not only address the long-standing clinical challenge of identifying reliable predictors of NCIT response but also uncover novel biological mechanisms that connect TME characteristics to immunotherapy sensitivity, providing both practical clinical tools and theoretical insights for ESCC precision treatment.

### 4.1. Clinical Significance of the Predictive Model

The integrated Model A, which combines the ECiT score with clinical variables (clinical T stage and P53 status), achieved an AUC of 0.897 in the training cohort and 0.809 in the temporal validation cohort. This performance outperformed both the single-modality pathomic Model C (validation AUC = 0.751) and the clinical Model B (validation AUC = 0.624), confirming the additive value of multimodal data fusion in NCIT response prediction. The above results are based on the analysis of preoperative pathological biopsy images. Compared to radiomics-based approaches that predict responses using medical imaging [35,36], our model offers a key advantage by utilizing pretreatment biopsy samples to predict NCIT response prior to therapy initiation, which can provide a reference for the clinical need of initial treatment decision-making. Shi et al. [35] developed a 2D radiomics signature based on post-NICRT (neoadjuvant immunotherapy plus chemoradiotherapy) CT scans to predict pCR (validation AUC = 0.89), which is valuable for identifying candidates for active surveillance. Gao et al. [36] proposed a habitat-aware radiomics framework integrating 2.5D deep learning from baseline PET/CT to predict chemoimmunotherapy response (test AUC = 0.824), providing insights into resistance-linked subregions such as the invasive front. While these radiomics models excel at capturing macroscopic tumor features and spatial heterogeneity, they are inherently limited in directly visualizing the cellular TME. Future work could explore integrating CT-based radiomics and H&E-based pathomics into a comprehensive “radio-pathomic” model, potentially capturing both whole-tumor macroscopic heterogeneity and microscopic TME determinants to further refine precision treatment strategies for ESCC.

The inclusion of P53 status and clinical T stage in the integrated model further validates their biological and clinical relevance to NCIT efficacy. Our finding that P53 mutation is associated with a lower probability of NCIT response is consistent with previous reports of P53-mediated immunotherapy resistance in multiple cancer types [37] and extends this association to locally advanced ESCC. Additionally, clinical T3 stage was identified as an independent predictor of poor NCIT response, which may be attributed to the deeper tumor invasion in T3-stage lesions, leading to the limited penetration of therapeutic agents and impaired access of immune cells to the TME. Compared with conventional biomarkers such as PD-L1 (previously reported AUC = 0.610 for advanced ESCC [21]), the integrated model exhibits superior predictive accuracy, highlighting its potential to replace or complement existing markers in clinical practice. This finding is consistent with the work of Captier et al. [38], who demonstrated that integrating multi-omics data (including clinical and pathological data) in metastatic NSCLC yields multimodal predictive metrics.

### 4.2. Biological Insights into Pathomic Signatures

Mechanistic analyses using data from TCGA and GEO revealed that ESCC cases with high ECiT scores were characterized by enhanced immune activation, including significantly higher infiltration of CD4^+^ memory T cells and elevated ESTIMATE immune scores. CD4^+^ memory T cells play a crucial role in maintaining long-term anti-tumor immune responses and mediating sustained sensitivity to immunotherapy [39,40], which provides a biological basis for the predictive ability of the ECiT score. In contrast, cases with a low ECiT score showed the upregulated expression of genes involved in the activation of the UPR pathway, an evolutionarily conserved stress response that restores ER homeostasis under adverse conditions but exhibits pro-tumorigenic and immunosuppressive properties when chronically activated [41,42]. ER stress and UPR activation have been widely demonstrated to induce immunosuppressive phenotypes in tumor cells and stromal components, thereby inhibiting T cell-mediated anti-tumor immunity and promoting resistance to chemotherapy, targeted therapy, and immunotherapy [43,44]. Our results extend these findings to ESCC, suggesting that the ER stress–UPR activation axis may be a key driver of NCIT resistance in low ECiT score patients, consistent with the central role of UPR in modulating tumor immunity across solid tumors [43].

A critical finding linking pathomic features to the ER stress–UPR axis is the role of EIF2S3, a component of the eukaryotic initiation factor 2 (eIF2) complex. EIF2S3 directly participates in PERK-eIF2α-ATF4 signaling, one of three canonical UPR pathways that modulates protein synthesis and stress adaptation [41,45]. As shown in Figure 7F, EIF2S3 exhibits significant correlations with three key pathomic features. KEGG enrichment analysis further confirmed EIF2S3 as a key component of the UPR pathway, while TCGA immune infiltration data demonstrated that high EIF2S3 expression correlates with reduced CD4^+^ memory T cell infiltration (Figure 8F). These observations collectively support a potential dual mechanism by which EIF2S3 may modulate NCIT response in ESCC through regulating the ER stress–UPR axis. These correlations are consistent with a model in which the ER stress pattern mediated by EIF2S3 is reflected in the pathological features captured by the ECiT score. For instance, CAS_846 and DEB_237 reflect the remodeling of the downstream TME. Therefore, the ECiT score may indirectly quantify the activity of the EIF2S3-mediated ER stress–UPR pathway through these morphological features, providing a non-invasive window for understanding the molecular drivers of the response to NCIT, which is consistent with the potential of UPR-targeted strategies in reshaping the TME and improving the efficacy of immunotherapy [43].

### 4.3. Innovation and Limitations

Our pathomic approach fills a critical gap in the existing literature by focusing specifically on NCIT response prediction in ESCC. Previous AI-based pathomic studies in ESCC, such as the ESCC-PS system developed by Li et al. [29], have demonstrated promising performance in predicting progression-free survival after immunotherapy (AUC = 0.904 when combined with PD-L1). However, these studies did not evaluate neoadjuvant treatment settings or integrate clinical variables, limiting their applicability to NCIT. Our study complements these efforts by showing that combining histopathological features with clinical correlates not only improves predictive accuracy but also enhances the clinical translatability of the model, as clinical variables (e.g., T stage, P53 status) are routinely collected in clinical practice. Additionally, the identification of EIF2S3 as a molecular link between pathomic features and treatment response distinguishes our work from prior studies, which focused primarily on predictive performance rather than mechanistic validation.

Despite these strengths, this study has several limitations that should be acknowledged. First, this study is a retrospective single-center study with a relatively small sample size, which may lead to potential selection bias, increase the risk of model overfitting, and limit the statistical power and generalizability of the results. Multi-center validation with larger sample sizes is therefore essential to confirm the model’s robustness. Second, the follow-up period was relatively short, with only 1-year recurrence-free survival data available, precluding the analysis of long-term outcomes such as 2-year or 5-year overall survival and disease-free survival, key endpoints for evaluating the clinical impact of NCIT. Third, PD-L1 expression data were not collected in this study, which prevented the direct comparison of the model’s performance with this widely used immunotherapy biomarker; future studies should integrate PD-L1 testing to assess whether combining the ECiT score with PD-L1 status further improves predictive accuracy. Fourth, while the Vahadane algorithm was used to correct for H&E staining batch effects, we did not quantify the residual impact of staining variability on model performance, which could affect the reliability of ECiT score calculations in real-world settings. Fifth, only patients with R0 resection were included in this study, which may introduce potential selection bias and limit the generalizability of the prediction model to the overall population with resectable locally advanced ESCC. Finally, our mechanistic cohort was not entirely limited to resectable locally advanced disease, which may restrict the generalizability of our findings, and the functional role of EIF2S3 in regulating ER stress, LD metabolism, and immune function was inferred from correlation rather than experimental validation, warranting further in vitro and in vivo studies to verify these mechanisms.

### 4.4. Future Research Directions

To address the limitations of current research and further advance the progress in this field, we plan to conduct multi-center studies with larger sample sizes to verify the effectiveness of the comprehensive model in different clinical settings, including different geographical regions and medical systems. These studies should be stratified based on key variables (such as scanner type, staining protocol, and immune checkpoint inhibitors) to ensure the robustness of the model in various clinical situations. Secondly, combining multi-omics data with pathological features and clinical features can further improve the predictive accuracy of the model and provide deeper mechanistic insights, as well as conduct basic research to explore the functional roles of EIF2S3 and UPR in regulating the tumor microenvironment immune response in ESCC and verifying them in vitro and in vivo. Finally, designing prospective clinical trials to evaluate whether the model can accurately predict the benefits of NCIT and the unsuitable population will ultimately achieve the goal of improving patient prognosis. Lastly, developing user-friendly software integrated with a clinical pathological workflow will help transform this model into routine clinical practice.

## 5. Conclusions

In summary, this study develops a robust integrated model combining pathomic features (ECiT score) and clinical variables (T stage, P53 status) that may help predict NCIT efficacy in ESCC, with favorable performance in preoperative biopsy specimens, which may serve as a potential auxiliary indicator to assist personalized treatment strategies. Mechanistically, the study identifies correlative evidence supporting that EIF2S3-mediated ER stress–UPR activation may be a key driver of NCIT resistance in ESCC, linking pathomic features to tumor microenvironment immunosuppression and generating a mechanistic hypothesis for the molecular mechanisms of immunotherapy sensitivity, which may inform the development of UPR-targeted combinatorial therapies.

## Figures and Tables

**Table 1:** Distributions of demographic and clinicopathological variables of patients with ESCC in the training set and the validation sets.

Variables	Total (%) * N = 104	Train (%) N = 74	Validation (%) N = 30	** *p*_Value
Age	63.76 ± 6.63	63.36 ± 6.58	64.17 ± 6.93	0.581
Sex				0.673
Female	20 (29.23)	15 (20.27)	5 (16.67)	
Male	84 (80.77)	59 (79.73)	25 (83.33)	
Smoking history				0.914
Never	32 (30.77)	23 (31.08)	9 (30.00)	
Ever	72 (69.23)	51 (68.92)	21 (70.00)	
Pretreatment TNM stage				0.697
II	4 (3.85)	2 (2.70)	2 (6.67)	
III	100 (96.15)	72 (97.30)	28 (93.33)	
Pretreatment T stage				0.714
T2	41 (39.42)	30 (40.54)	11 (36.67)	
T3	63 (60.58)	44 (59.46)	19 (63.33)	
Pretreatment N stage				0.211
N0-1	78 (75.00)	58 (78.38)	20 (66.67)	
N2	26 (25.00)	16 (21.62)	10 (33.33)	
ICI types				0.668
PD-1i	79 (75.96)	57 (77.03)	22 (73.33)	
PD-L1i	6 (5.77)	5 (6.76)	1 (3.33)	
CTLA4i	19 (18.27)	12 (16.22)	7 (23.33)	
NCIT cycles				0.486
1	1 (0.96)	1 (1.35)	0 (0.00)	
2	94 (90.38)	65 (87.84)	29 (96.67)	
3	9 (8.65)	8 (10.81)	1 (3.33)	
P53 status				0.160
Wild type	41 (39.42)	26 (35.14)	15 (50.00)	
Mutant	63 (60.58)	48 (64.86)	15 (50.00)	
MPR status				0.323
Non-MPR	58 (55.77)	39 (52.70)	19 (63.33)	
MPR	46 (44.23)	35 (47.30)	11 (36.67)	
1-year RFS rate	3 (2.88)	2 (2.70)	1 (3.33)	0.684

* N: the number of datasets; ** *p*_value: the probability value.

## Data Availability

TCGA cohort is available as open data via the Cancer Genome Atlas: https://portal.gdc.cancer.gov/projects/TCGA-ESCA (accessed on 10 July 2025). Other data that support the findings of this study are available from the corresponding author upon reasonable request. All original code developed for this research has been deposited in GitHub (https://github.com, accessed on 29 November 2025) and will be publicly accessible starting from the publication date.

## Supplementary Materials

The following supporting information can be downloaded at: https://www.mdpi.com/article/10.3390/curroncol33030136/s1, Figure S1: The distribution of features in Model C (pathomics feature model) across different NCIT response groups in Tongji hospital cohort. It presents box-and-scatter plots illustrating the distribution of five key features (1814_ESCC, 1893_ESCC, 846_CAS, 237_DEB, 790_LYM) between different NCIT response groups. Each subplot displays the median (central line), interquartile range (box), range (whiskers), and individual data points (scatter) for the feature in each group. Statistical significance (*p* value) for group differences is indicated within each subplot. Blue boxes represent Group 0 (non-MPR group), and yellow boxes represent Group 1 (MPR group); Figure S2: Distribution of features in Model C (pathomics feature model) between the Tongji Hospital cohort and the TCGA-ESCC cohort.It presents kernel density plots illustrating the distributions of five imaging features(237_DEB, 1814_ESCC, 790_LYM, 1893_ESCC, 846_CAS) in two datasets: Tongji hospital dataset (blue) and TCGA-ESCC dataset (orange). The overlapping regions of the distributions are shaded in gray. Kolmogorov–Smirnov (KS) test *p* values for the comparison of feature distributions between the two datasets are indicated in each subplot title, all showing significant differences (*p* < 0.0001).; Figure S3: Kaplan-Meier Survival Curves of Immune Active and Immune Resistant Groups. It depicts Kaplan-Meier survival curves comparing the overall survival of patients in the immune active group (blue curve) and immune resistant group (red curve). The log-rank test *p* value (0.980) and hazard ratio (HR) with 95% confidence interval (95% CI: 0.991, 0.491–2.002) are indicated, showing no statistically significant difference in survival between the two groups. The table at the bottom presents the number at risk for each group at different time points (in years); Figure S4: Distribution of stromal score and ESTIMATE score between immune resistance and immune active groups. It displays violin plots illustrating the distribution of stromal score (left panel) and ESTIMATE score (right panel) in immune resistance and immune active groups. The width of each violin represents the density of data points at different score values. Horizontal lines within violins denote median (solid) and quartiles (dashed). “ns” indicates no statistically significant difference between the two groups (*p* > 0.05); Figure S5: Protein-protein interaction (PPI) network of key genes associated with ECiT score-related pathological image features. This network illustrates the interactions between the top 10 candidate genes associated with pathological image features and the core genes of the unfolded protein response (UPR) pathway (EIF2S1, ATF4, HSPA5, EIF2AK3, DDIT3). Nodes represent individual proteins, with colors indicating functional clusters; edges represent experimentally validated or predicted interactions; Table S1: Comparison of Clinical and Pathological Characteristics Between Non-MPR and MPR Groups; Table S2: Performance Metrics of Different Classification Models on Train and Validation Sets; Table S3: Univariate and Multivariate Logistic Regression Analysis in the Tongji Hospital Training Dataset; Table S4: erformance Metrics of Three Models for Predicting NCIT Response in Different Datasets; Table S5: TMB Calculation and Group Comparison Analysis in the TCGA-ESCC Cohort; Table S6: Marker genes for T cell subsets; Table S7: Kaplan-Meier survival analysis results of the ER/UPR pathway top 10 key differential genes associated with ECiT score-related pathological image.

## Abbreviations

AI	Artificial Intelligence
AUC	Area Under the Receiver Operating Characteristic Curve
CAS	Cancer-Associated Stroma
CNN	Convolutional Neural Network
DCA	Decision Curve Analysis
DEB	Necrotic Regions
DFS	Disease-Free Survival
DL	Deep Learning
EC	Esophageal Cancer
ESCC	Esophageal Squamous Cell Carcinoma
ESCCs	ESCC Cell Regions
ER	Endoplasmic Reticulum
GO	Gene Ontology
ICIs	Immune Checkpoint Inhibitors
KEGG	Kyoto Encyclopedia of Genes and Genomes
LYM	Tumor-Infiltrating Lymphocytes
LD	Lipid Droplets
MPR	Major Pathological Response
NCIT	Neoadjuvant Chemoimmunotherapy
NCRT	Neoadjuvant Chemoradiotherapy
NSCLC	Non-Small Cell Lung Cancer
OS	Overall Survival
pCR	Pathological Complete Response
ROC	Receiver Operating Characteristic Curve
SGD	Stochastic Gradient Descent
SNP	Single Nucleotide Polymorphism
TCGA	The Cancer Genome Atlas
TMB	Tumor Mutational Burden
TME	Tumor Microenvironment
TILs	Tumor-Infiltrating Lymphocytes
TLS	Tertiary Lymphoid Structures
TAMs	Tumor Associated Macrophages
WSIs	Whole-Slide Images
UPR	Unfolded Protein Response
GSEA	Gene Set Enrichment Analysis
GEO	Gene Expression Omnibus
ES	Enrichment Score
