# Supplementary material for: Deep Learning-Derived Pathomic Features Predict NCIT Efficacy in Resectable Locally Advanced ESCC: Clinical Utility and Mechanistic Insights
Source: Curr Oncol. 2026 Feb 26;33(3):136. doi: 10.3390/curroncol33030136 (PMC13025008; doi:10.3390/curroncol33030136)
Supplement: Supplementary file 1 [file curroncol-33-00136-s001.zip › curroncol-4044787-supplementary.pdf]

Supplementary Information for

Deep Learning-Derived Pathomic Features Predict NCIT Efficacy in Resectable Locally Advanced ESCC: Clinical Utility and Mechanistic Insights

This file includes:

1. **Supplementary Tables**

2. **Supplementary Figures**

Table S1. Comparison of Clinical and Pathological Characteristics Between Non-MPR and MPR Groups

Variables	Total (%) *N = 104	Non-MPR(%) N = 58	MPR (%) N =46	Statistic	*P* value
ECiT score	0.45 (0.28, 0.60)	0.32 (0.22, 0.50)	0.58 (0.45, 0.67)	Z=-5.30	<.001
Age	63.76 ± 6.63	63.36 ± 6.58	64.17 ± 6.93		0.581
Sex				χ^2=0.33	0.563
Female	20 (19.23)	10 (17.24)	10 (21.74)		
Male	84 (80.77)	48 (82.76)	36 (78.26)		
Smoking History				χ^2=3.16	0.076
Never	32 (30.77)	22 (37.93)	10 (21.74)		
Ever	72 (69.23)	36 (62.07)	36 (78.26)		
Pretreatment TNM stage				χ^2=0.08	0.782
II	4 (3.85)	3 (5.17)	1 (2.17)		
III	100 (96.15)	55 (94.83)	45 (97.83)		
Pretreatment T stage				χ^2=7.69	0.006
T2	41 (39.42)	16 (27.59)	25 (54.35)		
T3	63 (60.58)	42 (72.41)	21 (45.65)		
Pretreatment N stage				-	0.078
N0	3 (2.88)	1 (1.72)	2 (4.35)		
N1	75 (72.12)	38 (65.52)	37 (80.43)		
N2	26 (25.00)	19 (32.76)	7 (15.22)		
ICI types				-	0.159
PD-1i	79 (75.96)	40 (68.97)	39 (84.78)		
PD-L1i	6 (5.77)	5 (8.62)	1 (2.17)		
CTLA4i	19 (18.27)	13 (22.41)	6 (13.04)		
NCIT Cycles				-	1.000
1	1 (0.96)	1 (1.72)	0 (0.00)		
2	94 (90.38)	52 (89.66)	42 (91.30)		
3	9 (8.65)	5 (8.62)	4 (8.70)		
p53 status				χ^2=12.83	<.001
Wild type	41 (39.42)	14 (24.14)	27 (58.70)		
Mutant	63 (60.58)	44 (75.86)	19 (41.30)		
1-year RFS rate	3 (2.88)	3 (5.17)	0 (0.00)		0.684

t: t-test, Z: Mann-Whitney test, χ^2: Chi-square test, -: Fisher exact

SD: standard deviation, M: Median, Q_1: 1st Quartile, Q_3: 3st Quartile

Table S2. Performance Metrics of Different Classification Models on Train and Validation Sets

Molder name	Train set	Validation set			
	AUC	AUC	Accuracy	Recall rate	F1 score
LR	0.856	0.751	0.700	0.364	0.471
NB	1.000	0.727	0.667	0.455	0.500
XGB	1.000	0.699	0.600	0.273	0.333
RF	0.899	0.679	0.667	0.364	0.444
KNN	0.921	0.670	0.633	0.273	0.353
SVM	0.960	0.636	0.633	0.273	0.353
DT	1.000	0.512	0.600	0.182	0.250

Table S3. Univariate and Multivariate Logistic Regression Analysis in the Tongji Hospital Training Dataset

Variables	Univariate Analysis					Multivariate Analysis				
	β	S.E	Z	*P*	OR (95%CI)	β	S.E	Z	*P*	OR (95%CI)
Sex										
Female					1.00 (Reference)					
Male	-0.30	0.58	-0.52	0.601	0.74 (0.24 - 2.30)					
Pretreatment clinical TNM stage										
II					1.00 (Reference)					
III	-0.11	1.43	-0.08	0.938	0.89 (0.05 - 14.86)					
Smoking History										
Never					1.00 (Reference)					
Ever	0.75	0.52	1.44	0.151	2.11 (0.76 - 5.84)					
Pretreatment clinical T stage										
T2					1.00 (Reference)					1.00 (Reference)
T3	-1.35	0.50	-2.70	**0.007**	0.26 (0.10 - 0.69)	-1.43	0.67	-2.14	**0.032**	0.24 (0.07 - 0.89)
Pretreatment clinical N stage										
N0-1					1.00 (Reference)					
N2	-1.67	0.69	-2.42	**0.016**	0.19 (0.05 - 0.73)					
P53 status										
Wild type					1.00 (Reference)					1.00 (Reference)
Mutant	-1.99	0.56	-3.56	**<.001**	0.14 (0.05 - 0.41)	-1.47	0.69	-2.12	**0.034**	0.23 (0.06 - 0.89)
ECiT score	7.92	1.79	4.42	**<.001**	2754.27 (82.20 - 92287.55)	7.80	2.12	3.68	**<.001**	2441.78 (38.13 - 156383.42)
Age	-0.03	0.04	-0.84	0.399	0.97 (0.90 - 1.04)					

β: The regression coefficient; S.E The standard error; Z: Z-statistic; P: The probability value; OR (95% CI): The odds ratio with 95% confidence interval; Reference: The reference category for categorical variables.

Table S3 (Continued). Univariate and Multivariate Logistic Regression Analysis in the Tongji Hospital Training Dataset

Variables	Univariate Analysis				
	β	S.E	Z	*P*	OR (95%CI)
ICI types					
PD-1i					1.00 (Reference)
PD-L1i	-1.49	1.15	-1.30	0.194	0.23 (0.02 - 2.14)
CTLA4i	-0.80	0.67	-1.20	0.231	0.45 (0.12 - 1.66)
NCIT Cycles					
1					1.00 (Reference)
2	15.47	1455.40	0.01	0.992	5249770.47 (0.00 - Inf)
3	15.57	1455.40	0.01	0.991	5757812.78 (0.00 - Inf)

β: The regression coefficient; S.E The standard error; Z: Z-statistic; P: The probability value; OR (95% CI): The odds ratio with 95% confidence interval; Reference: The reference category for categorical variables.

Table S4. Performance Metrics of Three Models for Predicting NCIT Response in Different Datasets

**Model**	**Dataset**	**Accuracy**	**Sensitivity**	**Specificity**	**Precision**	**F1**
Model A	Train Set	0.851	0.800	0.897	0.880	0.836
	Valiation Set	0.767	0.909	0.684	0.603	0.741
Model B	Train Set	0.716	0.571	0.846	0.769	0.656
	Valiation Set	0.600	0.636	0.579	0.467	0.538
Model C	Train Set	0.797	0.800	0.795	0.778	0.789
	Valiation Set	0.633	1.000	0.421	0.500	0.667

Table S5. TMB Calculation and Group Comparison Analysis in the TCGA-ESCC Cohort

**Variables**	**Total (n = 94)**	**ECiT Score low (n = 51)**	**ECiT Score high (n = 43)**	**Statistic**	***P***
TMB, M (Q_1, Q_3)	2.09 (1.61, 2.73)	2.11 (1.84, 2.68)	1.97 (1.50, 3.07)	Z=-1.02	0.309

Z: Mann-Whitney test; M: Median, Q_1: 1st Quartile, Q_3: 3st Quartile

Table S6. Marker genes for T cell subsets

T Cell Subset	Signature Geneset
T1: Effector CD8$^+$ T cell	CD8A, CD8B, GNLY, GZMB, PRF1, GZMA, KLRD1, CTSW, KIR2DL4, NKG7
T2: Effector CD4$^+$ T cell	TNF, IL2, CD4, CCL5, CD69, CAPG, ID2, GZMA, GZMB, CCL20, ALOX5AP, CD40LG, CLEC2B
T3: HSP-high T cell	HSPA1A, HSPA1B, DNAJB1, HSP90AA1, HSPB1, FOS, JUN, NR4A1, DUSP1
T4: IFN-high T cell	IFIT3, IFI6, IFIT1, MX1, ISG15, IFIT2, RSAD2, OASL, LY6E, STAT1
T5: Exhausted CD8$^+$ T cell	CXCL13, PDCD1, CTLA4, TIGIT, FOXP3, IL10, LAG3, ENTPD1, HAVCR2, LAYN, VCAM1, KRT86
T6: Naive/memory T cell	IL7R, CCR7, KLRB1, LTB, FTH1, LMNA, ANXA1, GZMK, CST7
T7: Regulatory T cell (Treg)	FOXP3, IL2RA, CTLA4, TNFRSF4, TNFRSF18, LAYN, TIGIT, IL1R2

Table S7. Kaplan-Meier survival analysis results of the ER/UPR pathway top 10 key differential genes associated with ECiT score-related pathological image

Genes	Log-rank P Value	HR(95%)
EIF2S3	**0.028**	0.429 (0.198 - 0.931)
SSR1	0.726	0.877 (0.419 - 1.834)
SELENOS	0.234	1.556 (0.746 - 3.247)
RPN2	0.731	1.137 (0.546 - 2.370)
EDEM2	0.275	0.659 (0.310 - 1.401)
MAP2K7	0.469	1.319 (0.622 - 2.801)
SEC23B	0.782	0.903 (0.436 - 1.869)
RRBP1	0.422	1.341 (0.653 - 2.754)
LAMN1	0.788	1.112 (0.532 - 2.324)
PDIA3	**0.037**	2.244 (1.030 - 4.888)

2. Supplementary Figures

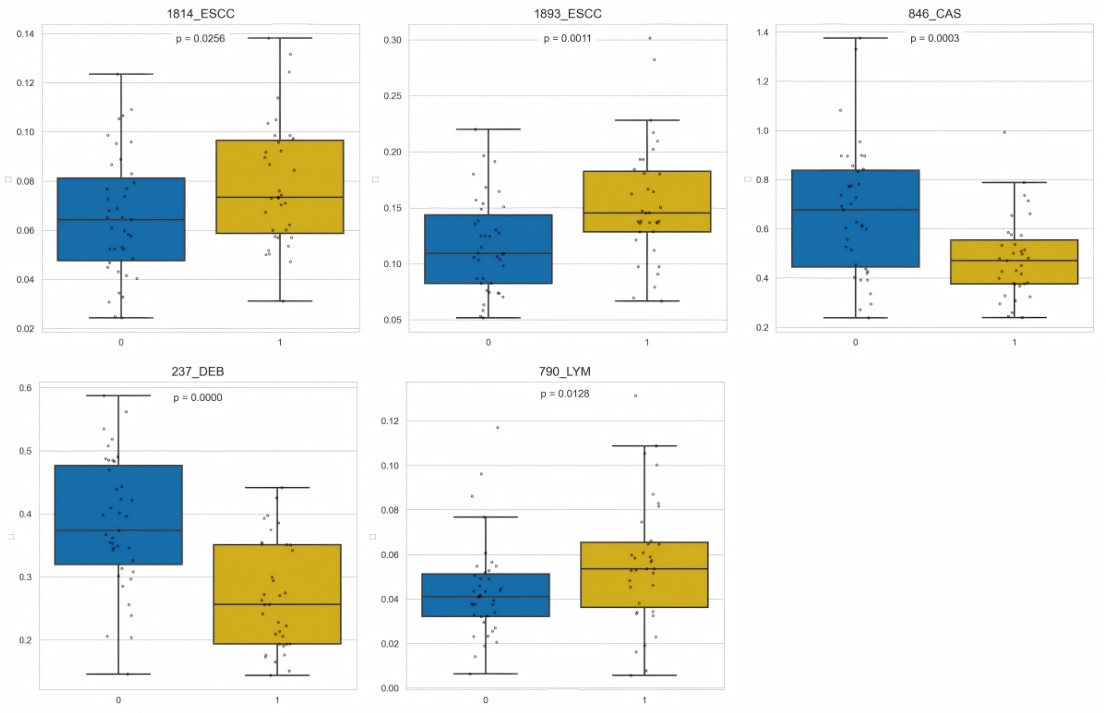

Figure S1. The distribution of features in Model C (pathomics feature model) across different NCIT response groups in Tongji hospital cohort. It presents box-and-scatter plots illustrating the distribution of five key features (1814_ESCC, 1893_ESCC, 846_CAS, 237_DEB, 790_LYM) between different NCIT response groups. Each subplot displays the median (central line), interquartile range (box), range (whiskers), and individual data points (scatter) for the feature in each group. Statistical significance (*P* value) for group differences is indicated within each subplot. Blue boxes represent Group 0 (non-MPR group), and yellow boxes represent Group 1 (MPR group).

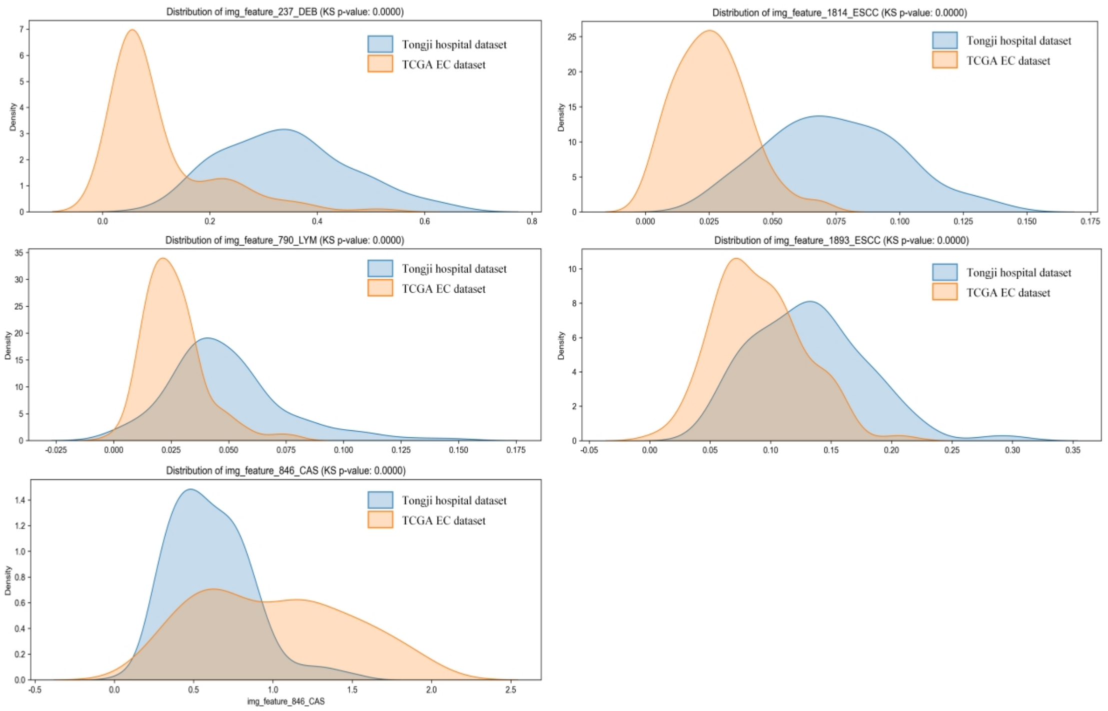

Figure S2. Distribution of features in Model C (pathomics feature model) between the Tongji Hospital cohort and the TCGA-ESCC cohort.It presents kernel density plots illustrating the distributions of five imaging features(237_DEB, 1814_ESCC, 790_LYM, 1893_ESCC, 846_CAS) in two datasets: Tongji hospital dataset (blue) and TCGA-ESCC dataset (orange). The overlapping regions of the distributions are shaded in gray. Kolmogorov–Smirnov (KS) test *P* values for the comparison of feature distributions between the two datasets are indicated in each subplot title, all showing significant differences ($P < 0.0001$).

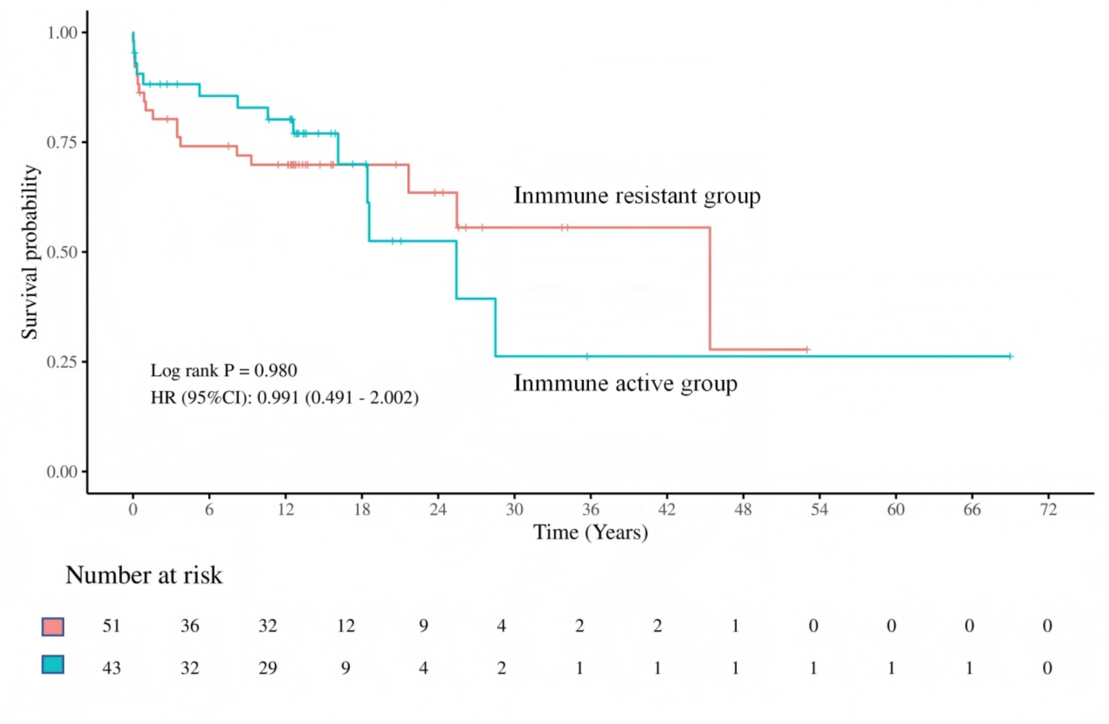

Figure S3. Kaplan-Meier Survival Curves of Immune Active and Immune Resistant Groups. It depicts Kaplan-Meier survival curves comparing the overall survival of patients in the immune active group (blue curve) and immune resistant group (red curve). The log-rank test *P* value (0.980) and hazard ratio (HR) with 95% confidence interval (95% CI: 0.991, 0.491-2.002) are indicated, showing no statistically significant difference in survival between the two groups. The table at the bottom presents the number at risk for each group at different time points (in years).

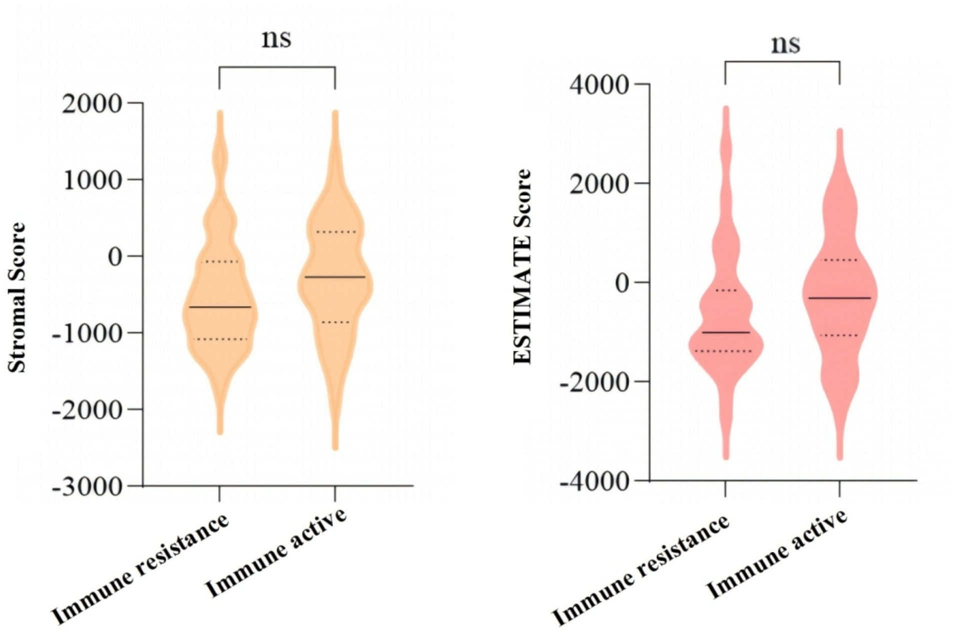

Figure S4. Distribution of stromal score and ESTIMATE score between immune resistance and immune active groups. It displays violin plots illustrating the distribution of stromal score (left panel) and ESTIMATE score (right panel) in immune resistance and immune active groups. The width of each violin represents the density of data points at different score values. Horizontal lines within violins denote median (solid) and quartiles (dashed). "ns" indicates no statistically significant difference between the two groups ($P > 0.05$).

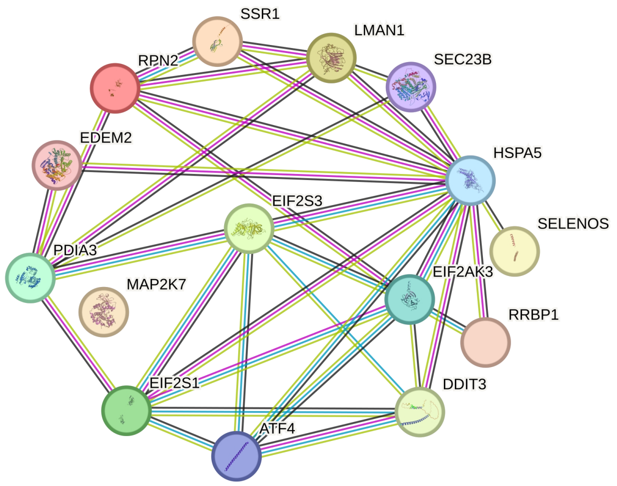

Figure S5. Protein-protein interaction (PPI) network of key genes associated with ECiT score-related pathological image features. This network illustrates the interactions between the top 10 candidate genes associated with pathological image features and the core genes of the unfolded protein response (UPR) pathway (EIF2S1, ATF4, HSPA5, EIF2AK3, DDIT3). Nodes represent individual proteins, with colors indicating functional clusters; edges represent experimentally validated or predicted interactions.
